# Three-Glass Test to Culture Prostate Secretion and Semen of Chronic Prostatitis Patients

**DOI:** 10.3390/diagnostics15131589

**Published:** 2025-06-23

**Authors:** Konstantinos Stamatiou, Hippocrates Moschouris, Konstantinos Tzelepis, Gianpaolo Perletti, Vittorio Magri

**Affiliations:** 1 Urology Department, Tzaneio General Prefecture Hospital, 18536 Piraeus, Greece; 2 Radiology Department, Tzaneio General Prefecture Hospital, 18536 Piraeus, Greece; hipmosch@gmail.com; 3 Urology Department, Agios Panteleimon General Hospital, 18450 Nikaia, Greece; 4 Department of Biotechnology and Life Sciences, University of Insubria, 21100 Varese, Italy; gianpaolo.perletti@uninsubria.it; 5 Urology Clinic, ASST Fatebenefratelli Sacco Hospitals, 20026 Milan, Italy

**Keywords:** chronic bacterial prostatitis, Meares–Stamey 4-glass test, 3-glass test, 2-glass test

## Abstract

**Background/Objectives**: Currently, the Meares–Stamey 4-glass and the 2-glass tests are used for diagnosing chronic prostatitis subtypes. Both tests include prostatic massage. Failure to extract prostatic secretions—for any reason—can result in a non-diagnostic test. Evidence from everyday practice and studies shows that expressed prostatic secretions are successfully recovered in less than 50% of the examined patients, and an important number of post-massage urine samples are missing prostatic secretions. This study evaluated a simpler test, the 3-glass (pre-ejaculation, ejaculation, and post-ejaculation) test. We compared it with the 4-glass and the 2-glass tests to detect inflammation and bacteria in men with chronic prostatitis symptoms. **Methods:** The study population included patients with chronic prostatitis symptoms. Subjects were assigned in each visit to undergo either the 4-glass, the 2-glass test, or the 3-glass test. The comparison among the three tests was based on the percentage of bacterial detection, the percentage of false-negative diagnoses, and the percentage of shifts among chronic prostatitis subtypes in the follow-up visits of recurrent patients. **Results:** A total of 157 patients were finally evaluated. Fifty-nine (59) patients underwent the 4-glass test (Group A), sixty-seven (67) underwent the 3-glass test (Group B), and thirty-one (31) underwent the 2-glass test (Group C). No statistically significant differences in the comparisons above were found. **Conclusions**: A comparison of the three diagnostic tests showed equivalence of the total ejaculate culture-based 3-glass test to the conventional prostatic secretion culture-based tests.

## 1. Introduction

Despite the recent progress in chronic, either bacterial or non-bacterial, prostatitis management, many cases relapse. The reasons remain unknown; however, they were mainly attributed to host, bacterial, and treatment-related factors. An ignored yet obvious reason could be misdiagnosis and misclassification among chronic prostatitis subtypes. Accumulating evidence suggests pitfalls and caveats in the diagnostic approach of chronic prostatitis to contribute to treatment failure [1]. Currently, the Meares–Stamey 4-glass test is the standard method of assessing inflammation and the presence of bacteria in the lower urinary tract in men. Since it has strong concordance with the 4-glass test, the 2-glass test (pre-massage and post-massage) was introduced as a reasonable alternative when expressed prostatic secretions are not obtained [2]. Both tests have value in diagnosis and treatment since they are also used for distinguishing between chronic prostatitis subtypes. Both tests include prostatic massage. This technique is performed by stroking the prostate several times to extract expressed prostatic secretions (EPSs). EPSs alone or mixed with urine (VB3/post-massage urine sample) are examined under a microscope. Failure to extract prostatic secretions can result in an non-diagnostic test. Evidence from everyday practice and studies shows that expressed prostatic secretions are successfully recovered in less than 50% of the examined patients, and an important number of post-massage urine samples are missing prostatic secretions [3,4]. In addition to the abovementioned pitfalls, most urologists do not use prostatic secretion culture-based tests in daily practice because of the time it takes and the difficulty in performing a prostate massage, and many patients refuse follow-up since both tests are somewhat awkward [5].

Post-ejaculation urine analysis can be a diagnostic tool for detecting chronic bacterial prostatitis (CBP). This test involves analyzing urine collected immediately after ejaculation to identify the presence of bacteria or inflammatory cells that may indicate an infection in the prostate gland. The post-ejaculation urine test differentiates between normal and abnormal results by analyzing the urine sample for specific markers, such as bacteria and white blood cells. The absence of these elements suggests no infection. The test results are interpreted alongside total ejaculation culture findings to confirm or rule out other conditions, like chronic non-bacterial prostatitis subtypes. Importantly, post-ejaculation urine culture is currently the only means to evaluate CBP in patients with retrograde ejaculation (e.g., patients with a history of TUR-P). In this study, we evaluated a simpler test, the 3-glass (pre-ejaculation, ejaculation, and post-ejaculation) test, and we compared it with the Meares–Stamey 4-glass and the 2-glass tests to detect inflammation and bacteria in men with chronic prostatitis symptoms. It should be mentioned that chronic prostatitis (CP) symptoms are not specific, and so they can overlap with other conditions. They usually include pain (persistent discomfort in the pelvic area and lower back, chronic scrotal and penile pain, difficulty urinating, frequent urination, a weak stream, painful ejaculation, and erectile dysfunction. Symptoms persist for at least three months before patients seek medical advice [6,7].

## 2. Methods

The study population included patients diagnosed with chronic prostatitis symptoms who presented themselves to the prostatitis clinic of the “Tzaneio” General Hospital between January 2023 and January 2025. Subjects were assigned in individual visits to undergo the 4-glass, the 2-glass, or the 3-glass tests.

The locally appointed Ethics Committee approved the research protocol 7312/12-12-22.

Eligible participants were randomized twice: at the time of an initial diagnosis and upon the follow-up visit. Upon every single visit, patients were asked to choose between a blue (Group 1), a red (Group 2), and a green (Group 3) closed envelope containing informative material and instructions regarding the chosen diagnostic procedure.

Inclusion criteria: To ensure accurate and relevant results, participants had to meet the following:

1. Diagnosis of chronic prostatitis: Patients must exhibit clinical signs and symptoms of prostatitis according to NIH criteria, applied both to category II Chronic Bacterial Prostatitis (CBP) and category III Chronic Prostatitis/Chronic Pelvic Pain Syndrome (CP/CPPS) (NIDDK Chronic Prostatitis Workshop, Bethesda, USA, 7–8 December 1995) [8]. These symptoms include scrotal, penile, and pelvic pain; urinary difficulties (nocturia and dysuria); or ejaculation disturbances.
2. Microbiological confirmation for category II (infection confirmed through urine, total ejaculate, or prostatic secretion cultures).
3. Microbiological confirmation for category IIIa (detection of pyuria in urine analysis).
4. Clinical confirmation for category IIIb (pain or discomfort during prostate palpation).
5. Age requirement: Adults aged 18 years and older.
6. Symptom Duration: Onset of symptoms within three months of the beginning of the study

Exclusion criteria: Patients with the following characteristics are not included to ensure accurate and relevant results.

1. Patients suffering from conditions affecting either bacterial virulence or host response (e.g., immunodeficiencies, abnormalities of the urogenital system, etc.) and individuals who received antibiotics or immunosuppressive treatment within 4 weeks of the first visit.
2. Patients with conditions like prostate cancer or severe benign prostatic hyperplasia.
3. Patients with recent ejaculation (less than 4 days before the visit).

Microbiological evaluation:

- The 4-glass Meares–Stamey test was based on the collection of a first-void urine sample (VB1, ~15 mL), followed by the collection of a midstream urine sample (VB2, ~15 mL). Prostate secretions were then obtained during a prostatic massage (EPS), followed by collection of a post-massage urine specimen (VB3, ~15 mL).
  This test was considered positive when (1) bacteria grew in the culture of the expressed prostatic secretion (EPS) and/or VB3 (post-prostate massage) urine sample and did not grow in VB1 (the first urinary output can collect any microorganisms present in the urethra, and, therefore, they represent an index of infection at that level) and VB2 (the mid-stream sample can collect microorganisms present in the bladder, therefore indicating infection at the level of the bladder); and (2) bacterial colonies in VB3 were at least ten-fold (one-log) higher than that of VB1 and VB2 urine samples.
- The 3-glass test consisted of the collection of pre-ejaculation urine, semen, and post-ejaculation urine.
  This test was considered positive when (1) bacteria grew in the culture of the total ejaculate (TE) sample and/or PoE (post-ejaculate) urine sample and did not in the PrE (pre-ejaculate) sample; and (2) bacterial colonies in the PoE were higher than that of the PrE urine sample.
- The 2-glass test.
  This test was considered positive when (1) bacteria grew in the culture of PoPM (post-prostate massage) urine sample and did not in the culture of the PrPM/PrE (pre-prostate massage) sample; and (2) bacterial colonies in PoPM were higher than those of PrPM urine sample.

Specimens were cultured undiluted in blood and MacConkey agar plates (Kallestad Lab., Austin, TX, USA), and then subjected to centrifugation for microscopic sediment examination. Evaluation of culture results was performed by two specialist microbiologists, who were not informed of the patient’s record. Identification was performed by conventional methods and the Vitek-2 Compact (bioMerieux, Craponne, France) system. Susceptibility testing was performed by disc diffusion and/or the Vitek-2 system. Interpretation of susceptibility results was based on Clinical and Laboratory Standards Institute (CLSI) guidelines.

Prevention of contamination of patient samples was based on optimal preparation of patients (abstaining from ejaculation for 2 to 5 days before the test, maintaining good hygiene by correct cleaning of the genital area, thorough washing of hands and glans penis with antiseptic soap before collecting the sample, disinfection of the glans penis with antiseptic solution performed by medical personnel, and use of a sterile container provided by the hospital laboratory). All samples were processed immediately after collection.

In addition, to avoid misinterpretation due to contamination of the various biological materials, a consensus threshold of 200 cfu/mL was defined for the diagnosis of chronic bacterial prostatitis in prostatic secretion culture-based tests [9].

According to the most recent trends, any bacteria can be the causative agent of bacterial prostatitis. Given that the condition often arises due to bacterial colonization of the urethra, *Staphylococcus species* are likely the causative agent of several chronic bacterial prostatitis cases. For this reason, they are considered the causative agent in symptomatic patients.

Clinical evaluation: Differentiation between chronic non-bacterial prostatitis subtypes (cat. III CP/CPPS of the inflammatory (type a) vs. the non-inflammatory (type b) subtypes) was based on the presence/absence of leukocytes in EPS/VB3/PoPM/PoE samples.

Several EPS/VB3/PoPM/PoE cultures were characterized as negative despite the presence of bacteria in the relative specimens. Possible explanations of the above finding include misinterpretation of test results, improper handling of specimens by the lab, delayed transport of the specimen to the laboratory, and urine culture specimens incorrectly labeled as contaminated. Since patients were symptomatic and did not receive antibiotics or immunosuppressive treatment within 4 weeks of their visit, this result could also be attributed to bacteria unable to grow in blood and MacConkey agar plates. These cases possibly represented false-negative cases [10]. They were indicated as showing “possible chronic bacterial prostatitis” and were excluded from the study, as they did not meet the inclusion criteria.

According to our findings, several cultures revealed higher bacterial colonies in the VB1, VB2, PrPM, and PrE compared to those of VB3, PoPM, and PoE samples. This finding indicates a simultaneous infection in both the prostate and the bladder. After careful evaluation of symptoms, clinical history, and diagnostic tests, they were considered mixed prostatitis/cystitis cases. The key criteria are as follows:

Symptoms: More prevalent symptoms, such as frequent urination, urgency, and burning sensation during urination.
History: The onset of chronic prostatitis symptoms precedes that of cystitis
Laboratory tests: A higher number of bacterial colonies in the VB1, VB2, PrPM, and PrE compared to that of VB3, PoPM, and PoE samples [11,12]. These cases were included in the study.

Patients who underwent the initial test received treatment according to UPOINT for cat III and the susceptibility test for cat II.

Cases diagnosed with different types of prostatitis upon follow-up or upon recurrence were reported as shifts between subtypes of chronic prostatitis and were attributed to the bias associated with each test.

The comparison among the three tests was based on the rate of bacterial detection, the rate of false-negative diagnoses, and the percentage of shifts among chronic prostatitis subtypes in the follow-up visits of recurrent patients.

Statistical analysis: Differences between proportions were analyzed with the Pearson’s Chi-square test, or Fisher’s exact test of significance for sample sizes smaller than 5. Analyses were performed in the “R 4.4.3” environment for statistical computation. The accepted significance level for the probability of a null hypothesis was <0.05.

## 3. Results

A total of 98 patients were enrolled in the study. Twenty-four met at least one of the exclusion criteria and did not participate in the study. The remaining 74 patients were randomized and underwent either the 4-glass, 3-glass, or 2-glass test. Fourteen patients were diagnosed with the chronic non-bacterial, non-inflammatory subtype; seven with the chronic non-bacterial, inflammatory subtype; and three with possible chronic bacterial prostatitis. Four were diagnosed with a condition different than than prostatitis and were discontinued from the study. Six out of the remaining patients were diagnosed with cystitis and chronic bacterial prostatitis, and 40 with pure chronic bacterial prostatitis.

Patients who underwent the initial test received treatment according to the UPOINT algorithm (cat III) and according to the susceptibility test (cystitis and chronic bacterial prostatitis, and pure chronic bacterial prostatitis cases).

According to the diagnostic test used to diagnose the above conditions, we formed three groups (Group A, 4-glass test; Group B, 3-glass test; and Group C, 2-glass test). No difference in median symptom severity and age among the patients of the three groups was found (Table 1).

Upon initial evaluation, bacterial detection (chronic bacterial prostatitis and mixed prostatitis/cystitis) was achieved in 51.8% of tests of Group A, 68.5% of tests of Group B, and 66.6% of tests of Group C (Table 2).

More precisely, upon initial evaluation, 13 patients in Group A (48.1% of the tests), 20 patients in Group B (57.1% of the tests), and 7 patients in Group C (58.3% of the tests) were diagnosed with chronic bacterial prostatitis (CBP). Similarly, one (11.1% of the tests), four (11.4%), and one (8.3%) patients in Groups A, B, and C, respectively, were diagnosed with cystitis and chronic prostatitis (C&CBP). Chronic non-bacterial, non-inflammatory subtype (CNBNI) was diagnosed in 22.2%, 14.2%, and 25% of the samples in Groups A, B, and C, respectively. Chronic non-bacterial, inflammatory subtype (CNBI) was diagnosed in 18.5% and 11.4% of the samples in Groups A and B, respectively. The percentage of false-negative diagnoses (possible chronic bacterial prostatitis/PCB) was 11.1% in the samples of Group A, 2.8% in Group B, and 8.3% in Group C (Table 2).

Regarding follow-up visits, only 17 patients chose an envelope of a different color from the one that they had chosen during the initial visit. These patients were examined with a different test during the following visit.

Seven patients did not attend the follow-up visit.

Symptom persistence and clinical remissions were detected in 10 patients. These were investigated in up to six visits.

Bacterial remissions and reinfections were detected in 9.3%, 28.5%, and 5.2% of the samples of Groups A, B, and C, respectively. Finally, shifts among chronic prostatitis subtypes were detected in 18.7%, 18.7%, and 26.3% of the samples of Groups A, B, and C, respectively. Table 3 provides a comparison of final diagnoses among the three groups.

In conclusion, 74 patients, examined over 157 visits, comprised the study material. Fifty-nine 4-glass tests (twenty-seven at initial evaluation and thirty-two at follow-up) were performed for Group A, sixty-seven new 3-glass tests (thirty-five at initial assessment and thirty-two at follow-up) were performed for Group B, and thirty-one 2-glass tests (twelve at initial evaluation and nineteen at follow-up) were performed for Group C.

Differences between proportions analyzed by both Pearson’s Chi-square test and Fisher’s exact test revealed no significant differences in bacterial detection when calculated on the total number of samples tested with each test (Table 4). 

From a microbiological viewpoint, the detected microorganisms in the prostatic fluid and the ejaculate practically do not differ (Table 5). However, the 3-glass test detected higher numbers of resistant pathogens compared to the 2- and 4-glass tests (*p*, 0.00014, Chi-square test, Table 6).

No statistically significant associations are found for individual pathogens. No significant results are found in the statistics that summarize the total number of Gram+ or Gram- on the total number of pathogens. However, the analyses performed on the total number of samples are significant:

1. The 3-glass test detects significantly higher proportions of Gram-negative pathogens, compared to both the 4-glass and the 2-glass tests (*p* = 0.014 and *p* = 0.005, respectively), when calculated on the total number of samples tested (Table 7).
2. The 3-glass test detects significantly higher proportions of total pathogens, compared to the 2-glass test (*p* = 0.02), when calculated on the total number of samples tested (Table 7).

## 4. Discussion

According to our findings, the bacterial detection rate (diagnoses of chronic bacterial prostatitis and mixed prostatitis/cystitis cases) was higher in Group B (3-glass test) than in Group A (4-glass test). However, the bacterial detection rate in Groups B and C (2-glass test) was similar. The percentage of false negative diagnoses (possible chronic bacterial prostatitis) was higher in Groups A and C than in Group B. Finally, the percentage of shifts among chronic prostatitis (CP) subtypes upon follow-up was relatively high in all three tests. The reasons explaining the abovementioned findings are probably the strict definitions used to classify CP syndromes following prostatic secretions culture-based tests. Traditionally, chronic bacterial prostatitis (CBP) is diagnosed by a 10-fold increase in bacteria in the EPS or VB3 specimens compared with VB1 and VB2 [13]. However, in a significant number of CBP cases in the present study, the increase in bacterial loads in VB3 specimens was between 2- and 3-fold compared to VB1 and VB2. In a similar number of cases, leucocyte counts in VB3 specimens were slightly higher than those in VB1 and/or VB2. These findings are identical to that of our previous study [4]. This criterion is somehow confounding given that white blood cell (leucocyte) counts have not been indicated to correlate with symptoms or the presence or absence of infection [14,15]. On the other hand, certain drawbacks, e.g., technical difficulties in performing prostatic massage (under the circumstances of obesity, rectal discomfort, or recent ejaculation) for which prostatic secretion cannot be obtained and a variety of factors (such as hyper-hydration, pollakiuria, and medications) that hide differences in leucocyte counts between pre- (VB1/VB2/prePM) and post-prostatic massage samples (VB3/PoPM) may shift the diagnosis to one or another direction.

Similar to our previous study, the most likely diagnosis following failure to extract prostatic secretions is that of chronic non-bacterial, non-inflammatory subtype [4]. Of note, in the present study, almost 60% of the 4-glass tests yielded sufficient quantities of EPS; therefore, in the remaining cases, the diagnosis was based only on VB3 cultures. In confirmation of the above, the detection rate of chronic non-bacterial, non-inflammatory subtype was significantly higher in Groups A and C than in Group B.

The difference in the rate of false-negative cases is probably due to the cut-off level of the number of bacterial colonies in both urine and prostate secretion samples. According to the literature, negative culture results may also occur for various reasons, including initiating empirical antibiotic therapy before obtaining the diagnostic test, high bacterial count cut-offs established by some laboratories (e.g., a threshold of 50,000 colony-forming units to report a test culture as “positive”), or insufficient sample volumes. On the other hand, the presence of fastidious organisms, anaerobic pathogens, or bacteria not detectable with the usual tests may explain cases characterized by false-negative cultures despite the actual presence of bacteria and no recent exposure to antibiotic intake reported [4].

As semen contains diluted prostatic material (around 20–30% of the total volume), together with material from the testes and other genital tract tissues, some authors have suggested the usefulness of semen analysis for detecting prostatic pathogens [16]. Although the culture of semen/total ejaculate for the diagnosis of bacterial prostatitis was introduced about 30 years ago [17], its usefulness and efficacy remain debated. The key factors that contribute to the continued discussion and assessment of semen culture for diagnosing bacterial prostatitis are as follows:

(1) Contamination: Semen samples can be easily contaminated by bacteria from the skin or surrounding environment, which can lead to misleading results and false positives. However, contamination of prostatic secretion can also occur during the collection process, which may similarly affect the accuracy of diagnostic tests.

(2) Inconsistent results: Studies and clinical experiences have shown varying levels of accuracy. Some studies report that total ejaculate cultures are reliable, while others find them less consistent in detecting the bacteria that cause prostatitis. In this study, the detection rate of the 3-glass test was higher than that of the 4-glass and 2-glass tests.

(3) Clinical relevance: The presence of bacteria in semen does not always correlate with the presence and severity of symptoms of prostatitis. This can make it challenging to determine whether the detected bacteria are indeed responsible for the patient’s condition, potentially leading to unnecessary treatments [18]. In fact, the ejaculate is a mixture of sperm from the testicles and fluids from the seminal vesicles, prostate, and bulbourethral glands. Therefore, the microbial flora in the ejaculate can include bacteria from the urethra, seminal vesicles, and prostate. In addition, seminal fluid is rich in fructose and other nutrients that support sperm and microbial viability. On the other hand, the prostatic fluid secreted by the prostate gland contains various enzymes, lipids, and citric acid. Although citric acid is a weak organic acid, it has natural antimicrobial properties and effectively inhibits the growth of bacteria, molds, and yeast [19]. Moreover, since most pathogenic bacteria prefer neutral or slightly alkaline conditions, the acidity of citric acid can hinder their growth. For the above reasons, some bacteria may be more prevalent in one type of fluid than the other. According to our findings, the microorganisms found in the ejaculate were similar to those found in prostatic fluid (*Escherichia coli*, *Enterococcus faecalis*, various species of Staphylococcus, etc.). Although these bacteria can form biofilms and adhere to surfaces, becoming resistant to antibiotics, in this study, most bacterial colonies were sensitive to most of the tested antibiotics. Of note, the resistance rate was found to be significantly higher in Group B (3-glass test) than in Groups A and C.

(4) Alternative methods: There are other diagnostic methods available, such as segmented urine cultures (Stamey–Meares 4-glass test and 2-glass test), which some healthcare providers prefer, due to their perceived higher reliability. However, some authors have suggested that segmented tests do not display sufficient sensitivity and may underestimate the prevalence of bacterial prostatitis cases, thus misdirecting the therapeutic approach to the disease [20,21]. Moreover, the coexistent urethral infection found in patients with CBP indicates the continuity of the infection within the genitourinary tract system, making the segmentation of urine cultures less important [22]. The total ejaculate culture test is much simpler to perform than the 4-glass or 2-glass tests and avoids the need for an unpleasant prostatic massage. A positive test diagnoses CBP even if the colony counts are low. However, a negative test does not necessarily rule it out [23,24]. Moreover, there is a misleading evaluation of chronic non-bacterial, inflammatory prostatitis due to the difficulty in distinguishing immature sperm from leukocytes [25]. Given that the distinction between CP syndromes (bacterial/non-bacterial and inflammatory/non-inflammatory types) is based on the presence or absence of bacteria and/or inflammatory cells in the TE and PoE samples, the abovementioned fact may shift the diagnosis in another direction [26].

As shown in this study, both the total ejaculate-based culture test and the prostatic secretion culture-based tests can play significant roles in CBP diagnosis; however, they suffer from different drawbacks and limitations. This was a real-life study, and the conditions in the ambulatory do not allow for a direct comparison of the three tests on the same patients without treatment. Patients demand a rapid investigation of their medical problems in order to receive treatment as soon as possible. For the above reason, we compared characteristics that may alter the results, such as age and NIH-CPSI, among the three three study groups to control biases. The fact that the findings from sperm cultures were comparable to those of EPS and post-PM cultures supports a supplementary role. Interestingly, Magri and associates found that the addition of microscopic examination and sperm culture to the standard Meares–Stamey test (five-glass test) increased the sensitivity 3.6 and 6.5 times more than the 4-glass test and the 2-glass test, respectively [23]. In light of a novel perspective of inflammation of the prostate gland, as part of an infection affecting the entire male genitourinary tract system, CBP patients should indeed undergo a comprehensive investigation to identify the underlying cause and appropriate treatment. This investigation typically includes the standard Meares–Stamey test (four-glass test), as well as Urethral Smear and Total Ejaculate Culture. These tests are crucial for diagnosing and managing infections in the male genitourinary tract system, ensuring that patients receive the appropriate care and treatment. On the other hand, chronic abacterial prostatitis, also known as chronic pelvic pain syndrome (CPPS), lacks a definitive diagnostic test, and the methodological quality of available studies of diagnostic tests is low [27]. This makes it challenging to establish reliable diagnostic criteria or treatment protocols [28].

## 5. Conclusions

A comparison of the three diagnostic tests showed equivalence of the total ejaculate-based culture test to prostatic secretion culture-based tests. However, the addition of total ejaculate culture to either the 4-glass or the 2-glass tests may improve their diagnostic accuracy. Criteria for differentiating types of prostatitis that render the interpretation of the culture results difficult could be revised.

## Figures and Tables

**Table 1:** Differences between both the mean age of patients age and the total score of the NIH-CPSI total score in cohorts tested with three different urinary tract segmented tests analyzed by Pearson’s Chi-square test.

	Mean Age	Mean NIH-CPSI
4-glass Meares–Stamey test	51.4	17.5
3-glass test	48.7	20.4
2-glass test	49.2	17.4
	*p* > 0.05	*p* > 0.05

**Table 2:** Initial evaluation: diagnoses per group.

Group A: 4-glass test	Diagnosis	Patients (*N*)	Tests (%)
*N* = 27	CBP	13	48.1
	CNBNI	6	22.2
	CNBI	5	18.6
	C&CBP	1	3.7
	PCB	1	3.7
	Other	1	3.7
Group B: 3-glass test	Diagnosis	*N*	%
*N* = 35	CBP	20	57.1
	CNBNI	5	14.3
	CNBI	4	11.4
	C&CBP	4	11.4
	PCB	1	2.9
	Other	1	2.9
Group C: 2-glass test	Diagnosis	*N*	%
*N* = 12	CBP	7	58.3
	CNBNI	3	25
	CNBI	0	0
	C&CBP	1	8.3
	PCB	1	8.3
	Other	0	0

CBP, chronic bacterial prostatitis; CNBNI, chronic non-bacterial, non-inflammatory; CNBI, chronic non-bacterial, inflammatory; C&CBP, cystitis and chronic prostatitis; PCB, possible chronic bacterial.

**Table 3:** Follow-up evaluation: diagnoses per group.

Group A: 4-glass test	T	U	CBP	CNBI	CNBNI	C&CBP	PCB
CBP	12	3			1		1
CNBI	1	2	1	2			
CNBNI	6		1				
C&CBP	2						
PCB	1						
Group B: 3-glass test	T	U	CBP	CNBI	CNBNI	C&CBP	PCB
CBP	9	5				1	
CNBI	4						1
CNBNI	2	1				1	1
C&CBP	4		1				
PCB	1		1				
Group C: 2-glass test	T	U	CBP	CNBI	CNBNI	C&CBP	PCB
CBP	6	1			2		
CNBI	2	1	1				
CNBNI							
C&CBP	2		1				
PCB	2		1				

T, treated. U, untreated. CBP, chronic bacterial prostatitis. CNBNI, chronic non-bacterial, non-inflammatory. CNBI, chronic non-bacterial, inflammatory. C&CBP, cystitis and chronic prostatitis. PCB, possible chronic bacterial. The number of diagnoses does not correspond to the exact number of patients since some were not treated during the first visit, and others were diagnosed with recurrence.

**Table 4:** Differences between bacterial detection rates in cohorts of patients tested with three different urinary tract segmented tests analyzed by Pearson’s Chi-square test.

	4GT	3GT	2GT	4GT vs. 3GT (*p*)	4GT vs. 2GT (*p*)	3GT vs. 2GT (*p*)
Patients with CBP (*N*)	16 (27.1%)	29 (43.2%)	14 (45.1%)	0.19	0.23	0.91
Total patients tested (*N*)	59	67	31			

**Table 5:** Pathogens (found alone or in combination) of each group.

4-Glass Test		3-Glass Test		2-Glass Test	
*Enterococcus faecalis*	13	*Escherichia coli*	25	*Enterococcus faecalis*	4
*Escherichia coli*	12	*Enterococcus faecalis*	17	*Escherichia coli*	4
*Staphylococcus epidermidis*	4	*Proteus mirabilis*	10	*Staphylococcus epidermidis*	3
*Staphylococcus hominis*	4	*Staphylococcus epidermidis*	9	*Staphylococcus hominis*	2
*Staphylococcus haemolyticus*	3	*Staphylococcus hominis*	4	*Staphylococcus haemolyticus*	1
*Klebsiella aerogenes*	2	*Streptococcus agalactiae*	3	*Klebsiella aerogenes*	1
*Staphylococcus lugdunensis*	1	*Haemophilus parainfluenza*	2		
*Pantoea* sp.	1	*Enterococcus Faecium*	1		
*Staphylococcus capitis*	1	*Klebsiella aerogenes*	1		
*Gonococcus*			1		

**Table 6:** Susceptibility rates in each group.

	4GT	3GT	2GT
Full sensitive	32	29	11
Any resistance	9	44	4
Total	41	73	15

**Table 7:** Pathogens detected with three different lower urinary tract segmented tests in patients diagnosed with chronic prostatitis. Significant results are shown in bold.

	Diagnostic Test						Significance of Difference Between Diagnostic Tests (Pearson’s *χ*^2^ or Fisher’s Exact Test)											
	**4-Glass **		**3-Glass **		**2-Glass **		**4G vs. 3G **	**4G vs. 2G **	**3G vs. 2G **	**4G vs. 3G **	**4G vs. 2G **	**3G vs. 2G **	**4G vs. 3G **	**4G vs. 2G **	**3G vs. 2G **			
**Species**	***n***	**%**	***n***	**%**	***n***	**%**	***p* vs. Gram-Positive**			***p* vs. Gram-Negative**			***p* vs. Total Pathogens**					
*Enterococcus faecalis*	13	30.23	17	24.64	4	28.57	0.65	0.99	0.75	**/**	**/**	**/**	0.62	0.99	0.76			
*Escherichia coli*	12	27.91	25	36.23	4	28.57	**/**	**/**	**/**	0.57	0.99	0.99	0.51	0.99	0.99			
*Proteus mirabilis*	0	0.00	10	14.49	0	0.00	n.a.	n.a.	n.a.	n.a.	n.a.	n.a.	n.a.	n.a.	n.a.			
*Staphylococcus epidermidis*	4	9.30	9	13.04	3	21.43	0.36	0.38	0.99	/	/	/	0.76	0.37	0.44			
*Staphylococcus hominis*	4	9.30	4	5.80	2	14.29	0.99	0.65	0.63	/	/	/	0.71	0.63	0.29			
*Staphylococcus haemolyticus*	3	6.98	0	0.00	1	7.14	0.99	n.a.	n.a.	n.a.	n.a.	n.a.	n.a.	n.a.	n.a.			
*Staphylococcus lugdunensis*	1	2.33	0	0.00	0	0.00	n.a.	n.a.	n.a.	n.a.	n.a.	n.a.	n.a.	n.a.	n.a.			
*Staphylococcus capitis*	1	2.33	0	0.00	0	0.00	n.a.	n.a.	n.a.	n.a.	n.a.	n.a.	n.a.	n.a.	n.a.			
*Streptococcus agalactiae*	3	6.98	0	0.00	0	0.00	n.a.	n.a.	n.a.	n.a.	n.a.	n.a.	n.a.	n.a.	n.a.			
*Klebsiella aerogenes*	1	2.33	0	0.00	0	0.00	n.a.	n.a.	n.a.	n.a.	n.a.	n.a.	n.a.	n.a.	n.a.			
*Haempophilus parainfluenzae*	0	0.00	2	2.90	0	0.00	n.a.	n.a.	n.a.	n.a.	n.a.	n.a.	n.a.	n.a.	n.a.			
*Enterococcus faecium*	0	0.00	1	1.45	0	0.00	n.a.	n.a.	n.a.	n.a.	n.a.	n.a.	n.a.	n.a.	n.a.			
*Pantoea* spp.	1	2.33	0	0.00	0	0.00	n.a.	n.a.	n.a.	n.a.	n.a.	n.a.	n.a.	n.a.	n.a.	**4G vs. 3G**	**4G vs. 2G**	**3G vs. 2G**
*Neisseria gonorrhoeae*	0	0.00	1	1.45	0	0.00	n.a.	n.a.	n.a.	n.a.	n.a.	n.a.	n.a.	n.a.	n.a.	***p* vs. total patients**		
**Total Gram-negative**	**14**	**32.56**	**38**	**55.07**	**4**	**28.57**	/	/	/	/	/	/	0.15	0.99	0.41	**0.014**	0.41	**0.005**
**Total Gram-positive**	**29**	**67.44**	**31**	**44.93**	**10**	**71.43**	**/**	**/**	**/**	**/**	**/**	**/**	0.21	0.90	0.31	0.85	0.32	0.39
**Total pathogens**	**43**	**100**	**69**	**100**	**14**	**100**										0.19	0.21	**0.02**
**Total patients**	**59**		**67**		**31**													

4G, 4-glass test; 3G, 3-glass test; 2G, 2-glass test; n.a., not assessable (one sample is zero).

## Data Availability

The data presented in this study are available on request from the corresponding author. The data are not publicly available due to privacy, legal and ethical reasons.

## Abbreviations

The following abbreviations are used in this manuscript:

EPS	expressed prostatic secretion
TE	total ejaculate
VB1	first-void urine
VB2	second-void urine
VB3	third-void urine
PoPM	post-prostate massage
PoE	post ejaculate
PrPM	pre-prostate massage
PrE	pre-ejaculate
